# Facial asymmetry in dogs with fear and aggressive behaviors towards humans

**DOI:** 10.1038/s41598-022-24136-2

**Published:** 2022-11-15

**Authors:** Marcello Siniscalchi, Serenella d’Ingeo, Michele Minunno, Angelo Quaranta

**Affiliations:** Animal Physiology and Behavior Unit, Department of Veterinary Medicine, University of Bari Aldo Moro, 70121 Bari, Italy

**Keywords:** Emotion, Social behaviour

## Abstract

There is now scientific evidence that, in dogs, distinctive facial actions are produced in response to different emotionally-arousing stimuli suggesting a relationship between lateralized facial expressions and emotional states. Although in humans, relationships between facial asymmetry and both emotional and physiological distress have been reported, there are currently no data on the laterality of dogs’ facial expressions in response to social stimuli with respect to canine behavioral disorders. The aim of the present work was to investigate the facial asymmetries of dogs with fear and aggressive behavior towards humans during two different emotional situations: (1) while the dogs were alone in the presence of their owners and (2) during the approach of an unfamiliar human being. Overall, our results demonstrated high levels of asymmetries in facial expressions of dogs displaying fear and aggressive behaviors towards humans indicating that measuring facial asymmetries in dogs could prove to be a useful non-invasive tool for investigating physiology-based behavioral disorders.

## Introduction

There is now significant evidence regarding the presence of lateralized emotional functioning in the brain throughout the animal kingdom^1^. In humans and other primates, the study of facial asymmetries associated with emotional expressions revealed a greater left-hemiface intensity during emotion processing^2–4^. Given that most facial muscles are innervated by the contralateral side of the brain, this finding supported the hypothesis of a right hemispheric dominance within emotion functioning (i.e. the right hemisphere hypothesis, “RHH”). The RHH is one of the most widely recognized hypotheses concerning the lateralized emotional functioning of the brain, which is widely shared by the scientific community^5^. An alternative hypothesis, the valence hypothesis (“VH”), postulates that negative and positive emotions and the display of related behaviors are controlled by the right and left hemisphere, respectively^6,7^. Moreover, a third hypothesis focuses on emotional responses in terms of approach-withdrawal behavior (i.e. the ‘approach-withdrawal model’ or ‘motivational direction model’). This model assumes that the left hemisphere is mainly involved in the processing of those emotions producing an approach motivation, whereas the right hemisphere is specialized in processing emotions related to withdrawal motivations^8^.

In dogs, lateralized cognitive processing of emotions is indirectly reflected by behavioral asymmetries (e.g., head orienting response^9,10^, nostril use^11^ and tail wagging^12,13^) in response to different emotional stimuli. Overall, the findings of these studies are in line with both the “valence” and “motivational direction” models highlighting a complementary specialization of the two sides of dogs’ brain where the right hemisphere is mainly involved in the expression of intense emotions (which elicits withdrawal behavioral responses) and the left hemisphere has a prevalent role in the analysis of positive emotional stimuli (which elicits approach behavior)^14^.

In terms of dog facial expressions, recent studies have revealed that distinctive facial actions are produced in response to different emotionally-arousing stimuli^15^ and have highlighted the influence of domestication in shaping the facial muscle anatomy of dogs, specifically for the muscles involved in facial emotional communication with humans^16^. In a recent behavioral study, Nagasawa and colleagues^15^ investigated the lateralized facial expressions of dogs upon reunion with their owners and during the interaction with unfamiliar stimulus (i.e. a human or an object). A significant right lateralization bias of facial muscle movements in response to avoidance-eliciting stimuli (non-social objects) was observed. Conversely, dogs showed more left eyebrow movement in the presence of their owner with respect to resting conditions, suggesting a relationship between lateralized facial expressions and an emotional state in dogs. In humans, facial asymmetry has been related to emotional, psychological^17^ and physiological distress^18^. In addition, a relationship between facial asymmetry and human mental disorders has been reported, including those related to the Autism spectrum^19^. To date, there are no data on the lateralization of dog facial expressions in response to social stimuli with respect to canine personality traits. The aim of the present work is that of investigating facial asymmetries in dogs with different behavioral responses toward humans during to two emotional situations: (1) while the dogs were in the presence of their owner alone and (2) during the approach of an unfamiliar human.

Furthermore, considering that humans are able to discriminate positive and negative expressions in dogs as accurately as those of human children^20^, with slight differences between dog breeds^21^, images of dogs were presented to human volunteers who were asked to rate the intensity of the emotions expressed (i.e. happiness, fear, anger, sadness, and neutral) in order to obtain an assessment of emotional asymmetry in dog facial expressions.

## Methods

### Dog facial expressions

#### Subject selection

Spontaneous facial expressions were obtained from subjects selected among a population of 60 dogs studied by the Department of Veterinary Medicine of the University of Bari through specialist consultation regarding fear and aggressive-related behavior displayed during social interaction with unfamiliar humans. During consultation, the assessment of dog behavior was carried out by veterinary behaviorists. Information regarding dog personality traits was also collected by means of a questionnaire that was administered to their owners^21^. A short version of the C-BARQ questionnaire was employed^22^. Specifically, three items were selected: “Stranger-directed aggression” (item 1), “Owner-directed aggression” (item 2) and “Stranger-directed fear” (item 3)^11^. The owners were asked to rate their dog’s behavior on a 5-point-scale (ranging from 0 to 4) in specific given situations when aggressive or fear-related behavior may occur (see Supplementary Table S1). Among the general sample, dogs showing no aggressive and fearful behavior towards their owners (item 2; mean ≤ 1) were included in the study. A further 10 adult dogs showing no behavioral issues towards humans were selected as a control group from a general population of volunteers taking part in behavioral studies at the Animal Physiology and Behavior Unit of the University of Bari. According to the response to unfamiliar humans, four behavioral groups of dogs were identified: Aggressive “A” (i.e. aggressive behavior shown), Fear “F” (i.e. fear behavior shown), Aggressive–Fear “A–F” (i.e. both fear and aggressive behaviors shown) and Control “C” (i.e. no fear or aggressive behaviors shown). Subjects displaying the most intense aggressive and fear-related behaviors during veterinary consultation and the highest score in the related items of the questionnaire were included in the final sample. Specifically, high scores (mean ≥ 3) for item 1 (i.e. stranger-directed aggression) were considered in the “A” group; high scores for item 3 (i.e. stranger-directed fear) in the “F” group; high scores for both item 1 and item 3 in the “A–F” group; whereas low scores (mean ≤ 1) for both item 1 and 3 were considered in the Control “C” group. A total of 36 subjects were thus included for the selection of facial expressions (9 subjects for each behavioral group). Subjects included 24 male and 12 females of various breeds, with ages ranging between 1 and 10 years. The four groups were balanced in terms of age.

#### Recording dog facial expressions

**Figure 1:**
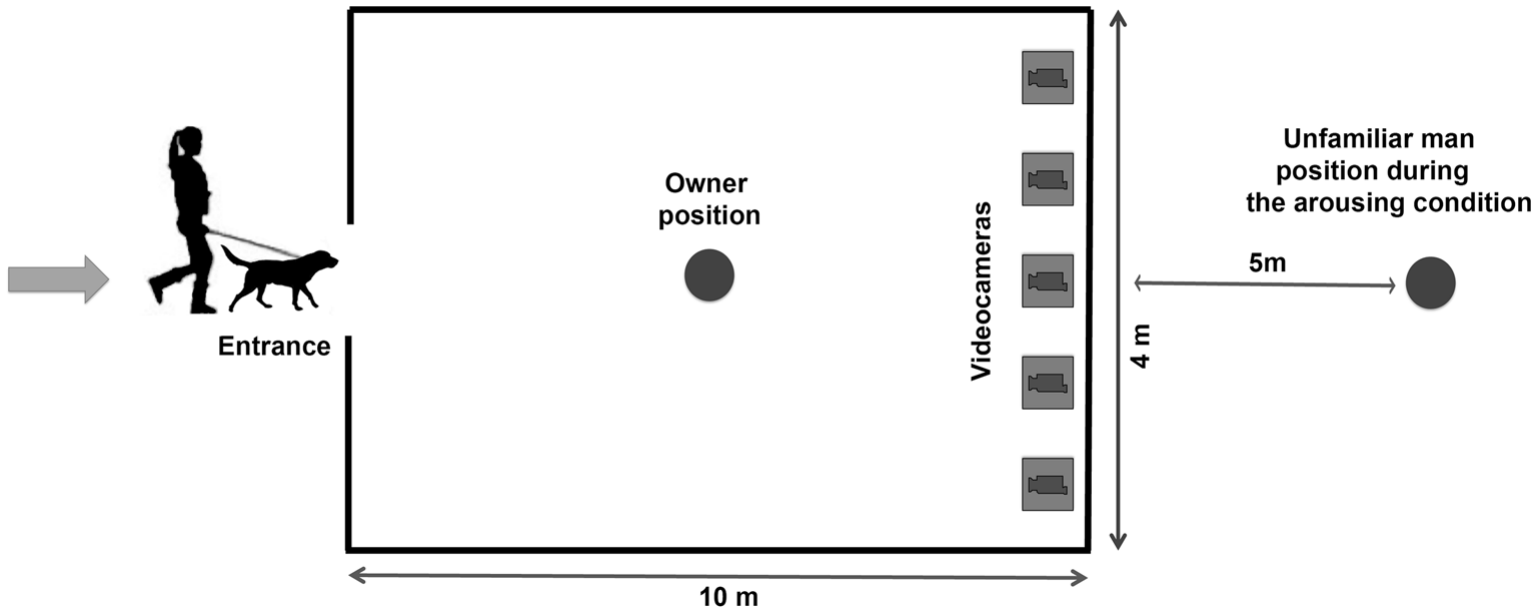
Experimental setup. Schematic representation of the experimental setup used for the recording of dog facial expressions.

The dogs and their owners entered an isolated outdoor area near the Department of Veterinary Medicine (a rectangular fenced area of around 40m^2^) and reached its center (Fig. 1). Each dog was then released and let free to move and explore the area for around 15 min in order to become familiar with their surroundings. Dogs showing high levels of stress (e.g. panting, freezing, sialorrhea^23^) during the familiarization phase were excluded from the final sample. Owners were asked to remain motionless and avoid any interactions with their dog (including visual and vocal signals). Five fixed cameras (Sony FDR-AX43®) were placed on tripods inside the fence and facing the owners and their dogs. Recordings started soon after the end of dog familiarization with the environment. Facial expressions were recorded during two social contexts: (1) while the dogs were in the fenced area alone with their owners (baseline condition); (2) in response to the approach of an unfamiliar male human who was the same for all the dogs (arousing condition), and who followed a standard procedure: he placed himself in front of the owner (and behind the cameras) outside the fence and 5 m from it; he then directly approached the dogs, along a straight line and staring at them (see Fig. 1). The approach occurred while the dogs spontaneously faced the cameras. Different dog-owner dyads were examined at weekly intervals in order to avoid contamination of dog odors in the fence influencing the other dogs’ behavior.

Three different observers with a high level of expertise in dog behavior and no prior knowledge of the study hypothesis carried out frame-by-frame analysis of video recordings in order to select the best images of dog facial expressions. Images of dogs facing the camera directly were preferred and, in case of more than one frame available, the clearest and most head-on and symmetric were chosen^3^. One picture for each subject in each context (i.e. in the presence of dogs’ owners alone, “baseline-condition”, and in response to the approach of an unfamiliar human, “arousing-condition”) was selected for the final sample. It included dogs whose related pictures were the clearest and head-on for both conditions in order to select four dogs for each behavioral group, which was balanced according to dog age (Aggressive: M = 5.50, S.D. = 2.38; Fear: M = 5.25; S.D. = 3.59; Aggressive–Fear: M = 5.25; S.D. = 3.30; Control: M = 5.50; S.D. = 2.89). The sample included 12 males (5 neutered) and 4 females (3 neutered) of various breeds (13 mix-breed, 1 Bullmastiff, 1 Border Collie and 1 Akita Inu), whose ages ranged between 1 and 10 years (M = 5.37; S.D. = 2.75).

#### Measuring asymmetries in dog facial expressions

**Figure 2:**
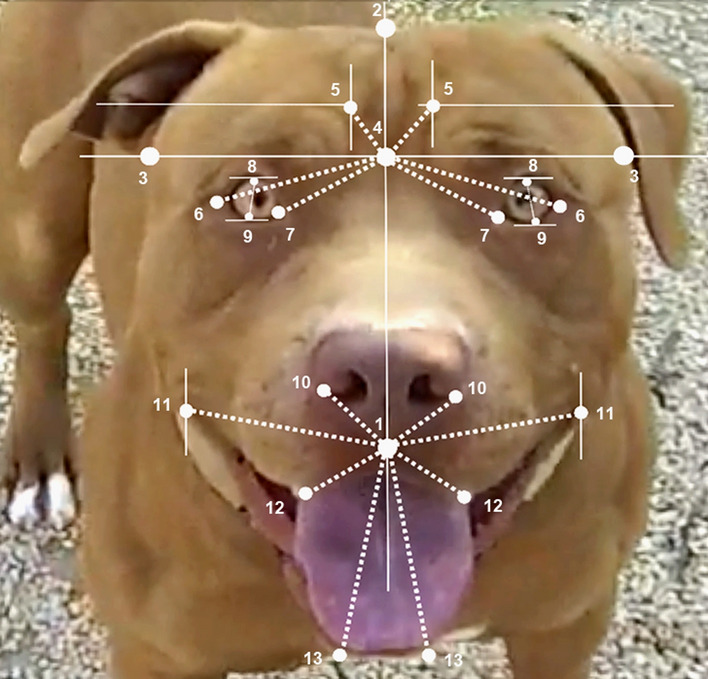
Facial landmarks. Twenty-three facial landmarks selected in order to calculate asymmetrical face ratio values.

Twenty-three facial landmarks (covering the eyebrows, eyes, noses and mouth) were selected in order to calculate facial asymmetrical ratio values (see Fig. 2). The asymmetry was measured along the vertical axis of the face. It was evaluated through the segment extending lengthwise through the base of the nose (1) and the tip of the head (2) (considering the center point of the ossis incisivum as the base of the nose and the crista sagittalis externa of the ossis frontale as the tip of the head). Pictures were vertically rotated until the vertical axis reached a 90^o^ angle with the horizontal line^3^. Subsequently, the vertical axis was intersected with a second perpendicular axis (horizontal axis) passing through the temporal bone's zygomatic process area (3) and a forehead mid-point was then obtained (4). The intersection between two lines tangent to the inner eyebrow and parallel to the vertical axis and the horizontal axis respectively was used in order to obtain the eyebrow landmark (5). Straight lines (dotted lines in Fig. 2) between landmarks were then obtained: eye brow—line between eyebrow landmark and forehead mid-point; eye width—line between lateral (6) and the medial cantus (7) from the forehead mid-point; eye height—line between the upper (8) and lower (9) part of the eyelid; nose—line between the outermost part of the nostrils (10) and the base of the nose (1); mouth width—line between the outermost part of the lip line (11) and the base of the nose (1) mouth height—segment between the lowest part of the upper (12) and lower (13) lip from the base of the nose (1). When the outermost part of the lip line was not detected clearly, mouth width landmark was not calculated. The length of each line was then identified using Adobe Photoshop Elite® and the ratio of the longest to the shortest line for each landmark of both right and left hemiface was calculated. The average value of all the ratios for each image was then calculated in order to obtain a facial asymmetry index. The rationale for the choice of this type of coefficient was based on using a calculation system sensitive to the asymmetry of dog faces as a whole: having used a coefficient analyzing the asymmetries of points at several levels, the calculation of the direction of the asymmetries (through formulas commonly employed for this type of directional calculation such as, for example: L − R/L + R where “L” signifies the average values of the landmarks of the left hemiface and “R” signifies the average values of the landmarks of the right hemiface) in even very asymmetrical faces could have nullified the result of the asymmetry itself. Following a series of measurements made on pilot tests it was observed that dogs expressing highly asymmetrical faces at different levels (e.g. at the levels of the eye line and of the mouth) but in a different directions, reduced or canceled the asymmetry coefficient of the face. It would therefore certainly be of interest in future studies to also analyze the direction of the asymmetries of different facial expressions at each different landmark level (e.g. eyebrows, eyes, nose, mouth etc.).

### Emotional expressions in chimeric dog faces as rated by humans

#### Participants

Given that there is little difference between the experienced and inexperienced in terms of the detection of basic facial expressions in dogs^20,24^, 32 untrained university students (i.e. they had received no prior training), 10 male and 22 female, participated in the study. They were between 18 and 32 years old (M = 21.3; S.D. = 3.39) and had no prior knowledge of the study hypothesis.

#### Visual stimuli

**Figure 3:**
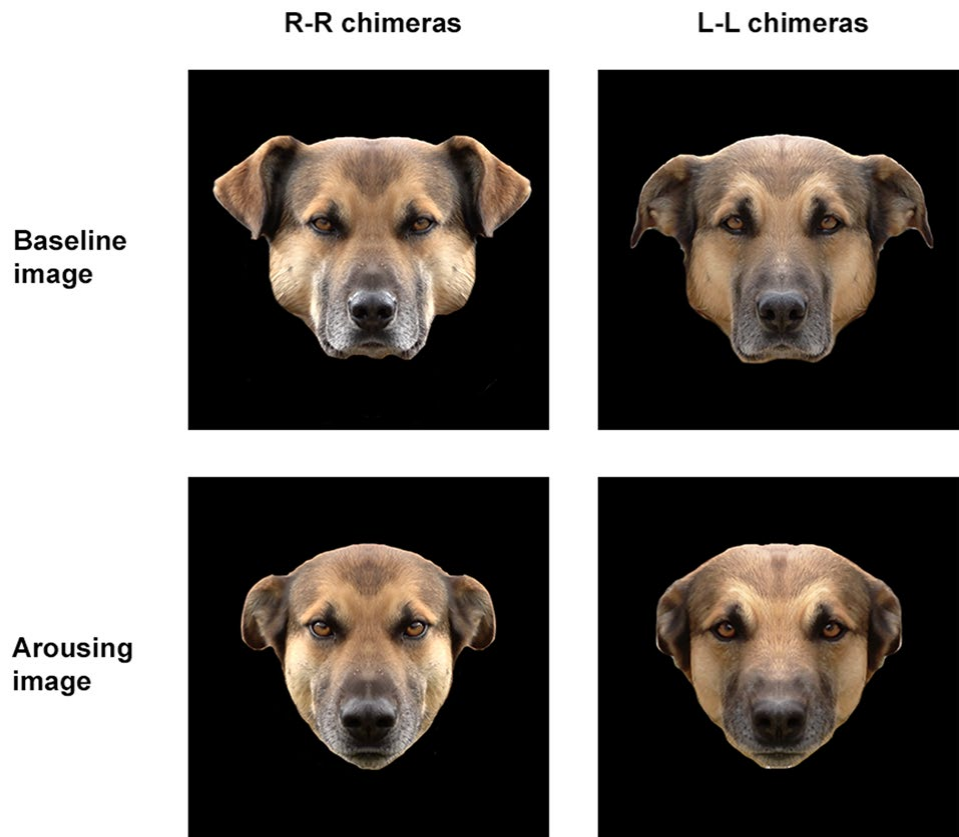
Chimeric pictures. Composite photographs displaying right-right (RR) and left-left (LL) chimeric facial expressions for baseline and arousing emotional conditions.

Pictures of dog facial expressions were edited using Adobe Photoshop Elite® in order to add a uniform black background. The vertical axis that was employed for identifying landmarks was here used to divide the face into two halves, the left and the right hemiface^3,4^. Each picture was then mirrored and paired with the original hemiface, thus obtaining a composite image (mirrored chimeric picture) displaying the right-right (RR) and left-left (LL) chimeric facial expressions. Two pictures for each emotional condition (i.e. baseline and arousing image chimeras) were thus obtained, representing the left and the right hemiface expression of the same emotional condition (see Fig. 3)^4^.

A total of 64 stimuli (2 chimeras × 2 emotional conditions × 16 subjects) were presented to the participants. The size of dog facial expressions was modified (i.e. enlarged or reduced) in order to homogenize it for all stimuli. Specifically, dog faces were inscribed within a rectangle (size 15.5 cm × 12.5 cm) so that the tip of the head and the chin were tangential to its bases. Moreover, picture brightness was balanced in the case of significant and relevant differences between the two chimeras.

#### Procedure

Dog facial expressions were presented to participants as a PowerPoint slideshow on a computer (SiComputer Nauta 01W PRO 15″®) in full screen mode and in a random order between subjects. Each volunteer seated in front of the computer and was verbally informed of the procedure. They were asked to rate the intensity of happiness, fear, anger, sadness, and neutral expressions for each picture on a Likert 5-point-scale (0- not at all; 1- slightly; 2- moderately; 3- very; 4- extremely). Each chimera appeared at the center of the screen where it remained for 10 s followed by a black background for a further 10 s to provide additional time to participants for completion of the questionnaire. In order to prevent volunteers’ fatigue, the 64 facial expressions were presented across two different sessions (32 pictures × each session) on the same day with a 20-min interval. Each picture was shown only once per each subject.

### Data analysis

#### Asymmetry of dog facial expression

The ratio calculated for the distance from the temporal bone's zygomatic process to the forehead mid-point in each image for the left and the right hemiface were analyzed using a one-sample t-test. This was done in order to determine whether on average the images were depicting dog faces that were head-on.

GLMM analysis was performed to assess the influence of “behavioral groups” (i.e. “A”, “F”, “A–F” and “C”) and the “experimental conditions” (and their interactions) (i.e. (1) while the dogs were alone in the presence of their owners and (2) during the approach of an unfamiliar human) on the test variables “facial asymmetry index”, with “subjects” as a random variable. Since the test variable values take a symmetric, bell-shaped distribution of a central (mean) value, “Normal” distribution and identity-link function were used to specify the target of the model. Bayesian information criterion (BIC) was used for selecting and comparing models based on the -2 log likelihood. To detect differences between different groups, Fisher’s Least Significant Difference (LSD) pairwise comparisons were performed.

#### Dog facial expressions rated by humans

GLMM analysis was performed in order to assess the influence of the “emotion category” (i.e. happiness, fear, anger, sadness, and neutral), “experimental conditions”, “chimeric faces” (i.e. RR chimera and LL chimera) and the “behavioral groups” (and their interactions) on the “emotional score” test variables, with “subjects” as a random variable. Since the test variable values were distributed along a positive scale that was skewed toward larger positive values, the inverse Gaussian distribution and identity-link function were used. Bayesian information criterion (BIC) was used for selecting and comparing models based on the -2 log likelihood. To detect differences between different groups Fisher’s Least Significant Difference (LSD) pairwise comparisons were performed.

Statistical analyses were performed using SPSS® software version 22 (IBM, Armonk, USA, NewYork).

### Ethics statement

The experiments were conducted according to the protocols approved by the Italian Minister for Scientific Research in accordance with EC regulations and were approved by the Department of Veterinary Medicine (University of Bari) Ethics Committee EC (Approval Number: 9/22). Written informed consent was obtained from all participants.

### Animal section

All experimental protocols involving the use of animals were approved by the Department of Veterinary Medicine (University of Bari) Ethics Committee EC (Approval Number: 9/22).

All tests were carried out in accordance with the guidelines approved by the Italian Minister for Scientific Research in accordance with EC regulations.

## Results

### Asymmetry in dog facial expressions

The analyses of the ratios calculated for the distance from the temporal bone's zygomatic process to the forehead mid-point in each image for the left and the right hemiface was not statistically significant, indicating the absence of bias toward a greater exposure of one side or the other of the dog faces (two-tailed one-sample t-test: t(31) = 1,213, *P* = 0.234; see Supplementary Table S2).

**Figure 4:**
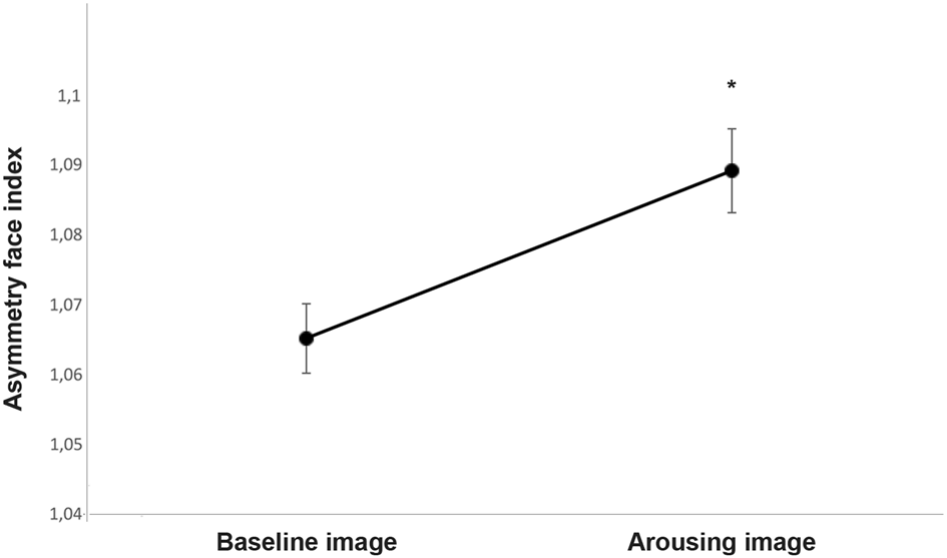
Facial asymmetry index. Facial asymmetry index of each dog during the two experimental conditions (group means with SEM are shown); Asterisks indicate significant biases. *P < 0.05; **P < 0.01 (Fisher’s LSD test).

**Figure 5:**
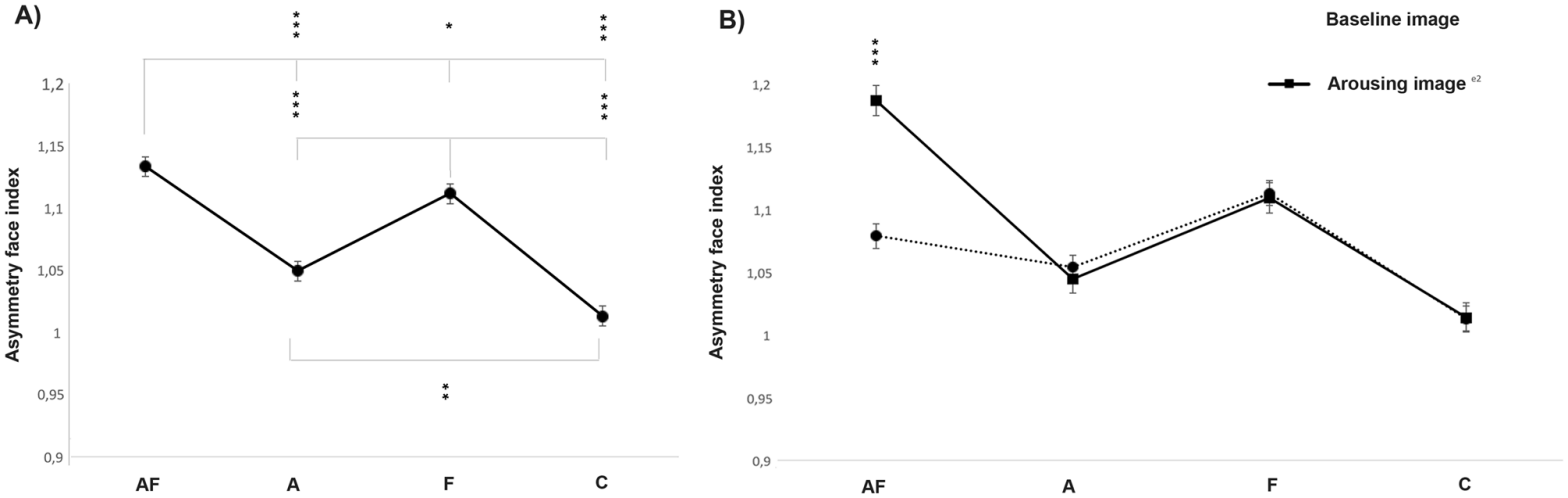
Facial asymmetry index and behavioral groups. (**A**) Facial asymmetry index of each behavioral group (“A”: dogs displaying high scores in stranger-directed aggression; “A–F”: dogs displaying high scores in both stranger-directed aggression and stranger-directed fear; “F”: dogs displaying high scores in stranger-directed fear; “C”: control dogs displaying low scores in both stranger-directed aggression and stranger-directed fear; group means with SEM are shown); Asterisks indicate significant biases. *P < 0.05; **P < 0.01 (Fisher’s LSD test). (**B**) Facial asymmetry index of each behavioral group during the baseline and arousing experimental conditions (i.e. group means with SEM are shown); Asterisks indicate significant biases. *P < 0.05; **P < 0.01 (Fisher’s LSD test).

The analysis of the facial asymmetry index revealed that dog faces were more asymmetric when subjects were approached by the unfamiliar human with respect to the baseline (presence of the owner alone (F(1, 24) = 9.919, *P* = 0.004; GLMM analysis); see Fig. 4). A statistically significant effect of the behavioral group was also observed (F(1, 24) = 53.359, *P* < 0.001; GLMM analysis; see Fig. 5A) indicating that subjects belonging to the “A–F” group had the highest level of asymmetry in their facial expressions, followed by “F” and then the “A” and “C” groups, respectively (“A–F” vs. “A” and “C” (*P* < 0.001); “A–F” vs. “F” (*P* = 0.046); “F” vs. “A” and “C” (*P* < 0.001); “A” vs. “C” (*P* = 0.002); pairwise comparisons). Finally, a behavioral groups × experimental conditions interaction was found (F(3, 24) = 13.978, *P* < 0.001; Fig. 5B): post hoc pairwise comparisons revealed that “A–F” dogs faces were more asymmetric when dogs were approached by the unfamiliar human with respect to the baseline (*P* < 0.001). No statistically significant differences were identified between the two different experimental conditions in the other groups (*P* > 0.05 in all comparisons).

### Emotional score

**Figure 6:**
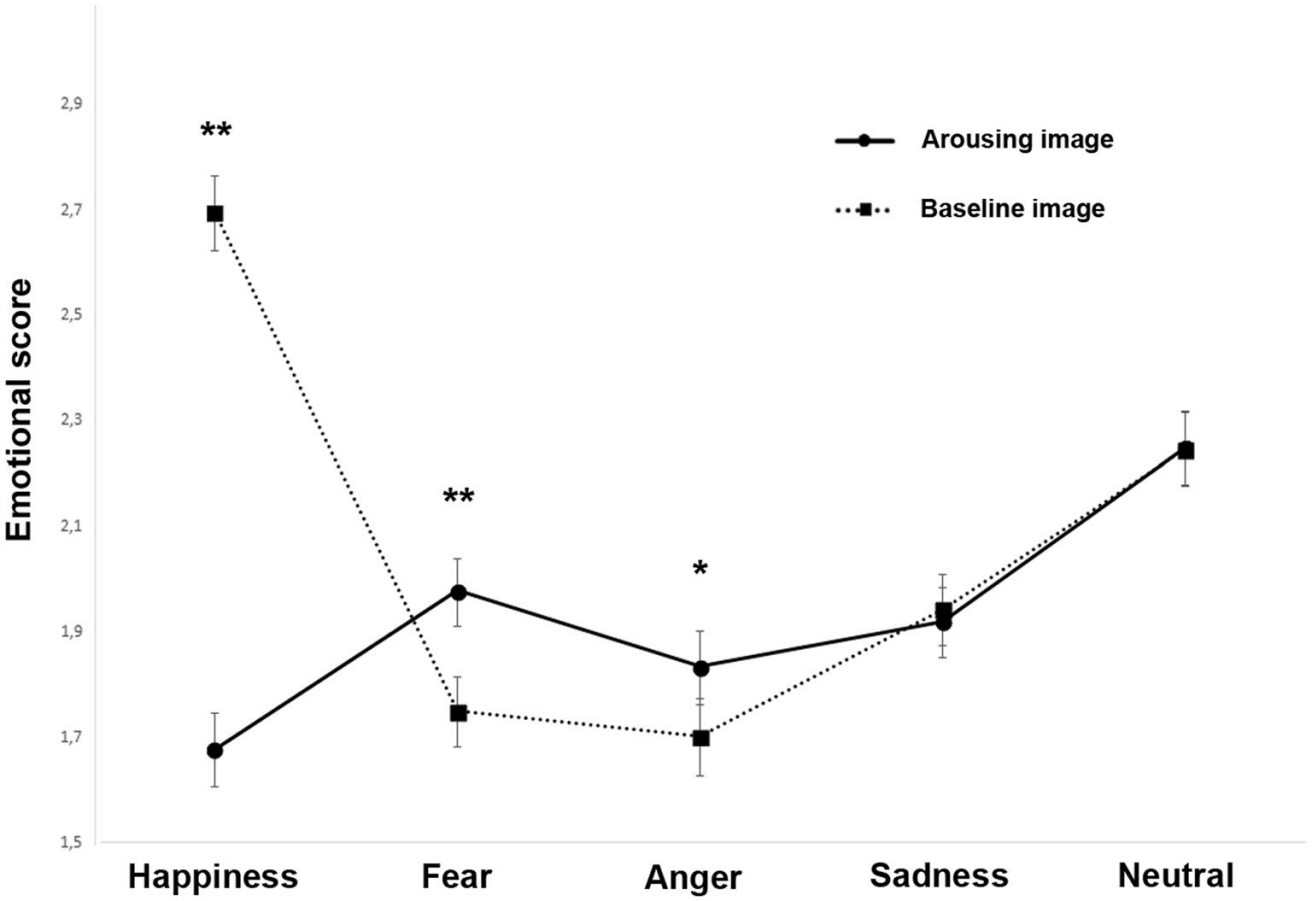
Emotional score (1). Dog facial expressions rated by humans for each emotional category (i.e. happiness, fear, anger, sadness, and neutral) during the baseline and arousing experimental conditions (group means with SEM are shown; Asterisks indicate significant biases. *P < 0.05; **P < 0.001; Fisher’s LSD test).

A significant main effect of the emotion category was observed (F(4, 4644) = 44.866, *P* < 0.001; GLMM analysis; see Supplementary Table S2): pairwise comparisons revealed that this main effect was due to scores for “happiness” and “neutral” being significantly higher than other emotions (*P* < 0.001). In addition, the score of “fear” (*P* = 0.020) and “sadness” (*P* < 0.001) were higher than “anger”. The GLMM analysis also revealed a significant main effect of the experimental conditions on the emotional score (F(1, 4644) = 25.878, *P* < 0.001) showing that dogs facial expressions received generally lower scores when subjects were approached by the unfamiliar human than in the baseline condition. A significant emotional score × experimental conditions interaction was also found (F(4, 4644) = 65.683, *P* < 0.001; GLMM analysis; Fig. 6): pairwise comparisons revealed that this main effect was due to scores for “happiness” being significantly higher (*P* < 0.001) at baseline than in response to the approach of the unfamiliar human; on the other hand, in the latter condition, dogs displayed higher scores for both “fear” (*P* < 0.001) and “anger” (*P* = 0.036) emotions.

A significant main effect was found for behavioral groups on the emotional score (F(3, 4644) = 8.333, *P* < 0.001): pairwise comparisons revealed that dogs belonging to the “A–F” and “F” behavioral groups had higher scores with respect to “A” and “C” groups (*P* = 0.001), whereas no statistical significant differences were found between “A–F” and “F” (*P* = 0.700) and between “A” and “C” (*P* = 0.966), respectively. A statistically significant interaction between emotion category and behavioral groups (F(12, 4644) = 19.580, *P* < 0.001) was revealed: the analysis demonstrated that “F” and “A–F” groups had higher scores for the “happiness” emotion with respect to “A” and “C” groups (*P* < 0.01 for all comparisons). Statistically significant differences were also found between “A” and “C” (*P* = 0.001) but not between “A–F” and “F” (*P* = 0.164). As for the “fear” emotion, analysis showed that the “A–F” and “F” groups had higher scores with respect to the “A” and “C” groups (*P* < 0.001 for all comparisons) and a statistically significant difference was identified between “A–F” and “F” (*P* = 0.013) but not between “A” and “C” (*P* = 0.171). As for “anger”, the “A–F” group revealed higher scores with respect to all other behavioral groups (“A–F” vs. “A”, *P* = 0.001; “A–F” vs. “C” and “F”, *P* < 0.001). No other statistically significant differences were identified (*P* > 0.05; pairwise comparisons). As for “sadness”, the analysis showed that the “C” and “F” groups had higher scores than the “A–F” and “A” groups (“C” vs. “A–F” and “A”, *P* < 0.001; “F” vs. “A–F” and “A”, *P* < 0.05). No other statistically significant differences were found (*P* > 0.05; pairwise comparisons). Finally, pairwise comparisons revealed that the “A” and “C” groups had higher scores for “neutral” emotions with respect to the “A–F” and “F” groups (*P* < 0.001). No other statistically significant differences were found (*P* > 0.05; for all other comparisons).

**Figure 7:**
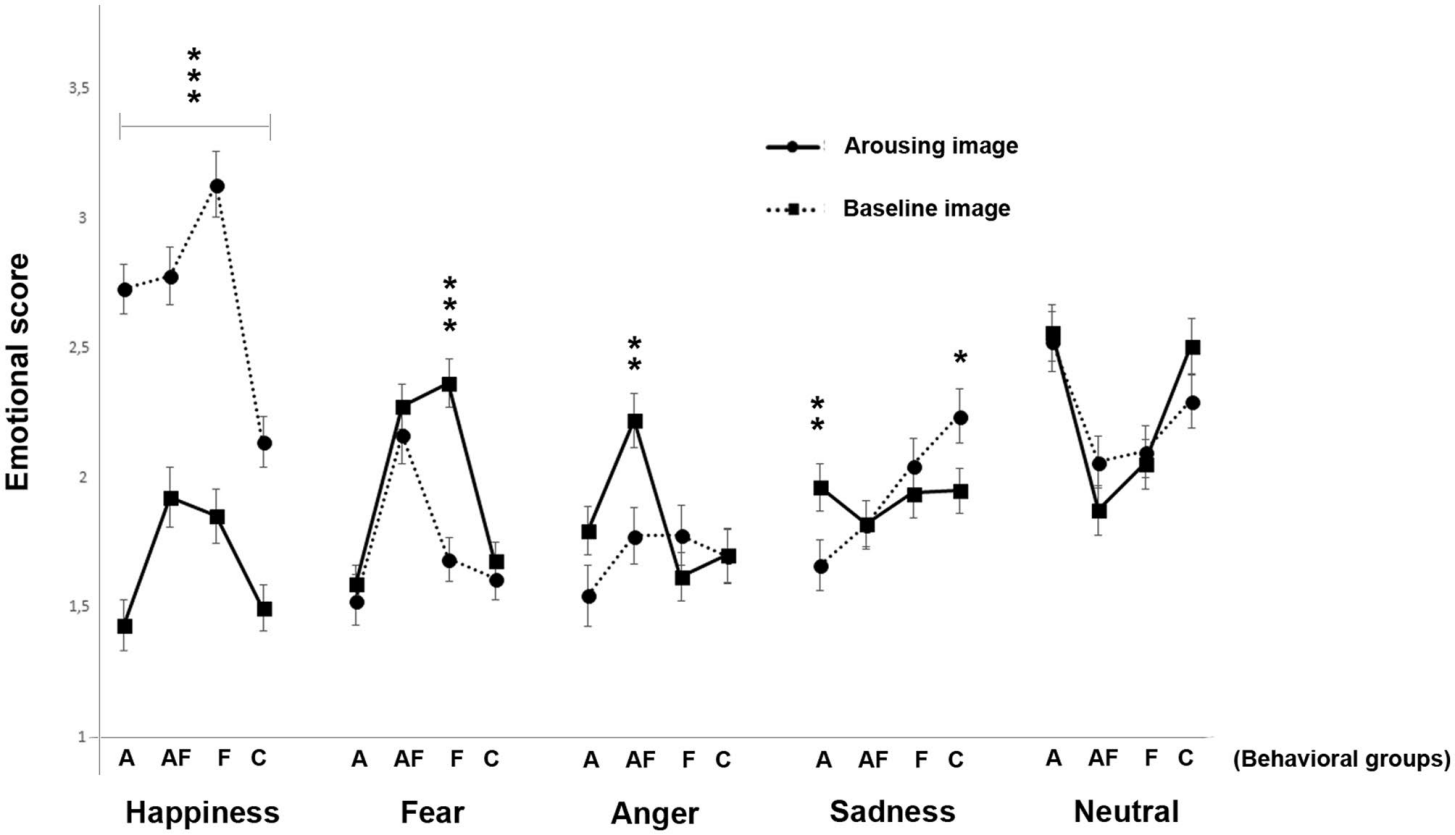
Emotional score (2). Dog facial expressions rated by humans for each emotional category (i.e. happiness, fear, anger, sadness, and neutral) of each behavioral group (“A”: dogs displaying high scores in stranger-directed aggression; “A–F”: dogs displaying high scores in both stranger-directed aggression and stranger-directed fear; “F”: dogs displaying high scores in stranger-directed fear; “C”: control dogs displaying low scores in both stranger-directed aggression and stranger-directed fear) during the baseline and arousing experimental conditions (group means with SEM are shown; Asterisks indicate significant biases. *P < 0.05; **P < 0.01; ***P < 0.001; Fisher’s LSD test).

A significant emotion category × experimental conditions × behavioral groups interaction was identified (F(12, 4656) = 6.983, *P* < 0.001; Fig. 7): pairwise comparisons revealed that the score for “happiness” was significantly higher at baseline for all the behavioral categories (*P* < 0.001); on the other hand, when dogs were in the presence of the arousing social stimulus, significantly high scores were found for “fear” emotions in the “F” group (*P* < 0.001) and for “anger” in the “A–F” group (*P* = 0.001). Finally, pairwise comparisons revealed high scores for “sadness” in the “A” group during the presence of the arousing social stimulus (*P* = 0.007) and an opposite bias in the “C” group (baseline condition > arousing stimulus: *P* = 0.010).

Statistically significant interaction between the emotion category and chimeric faces (F(4, 4644) = 2.865, *P* = 0.022) was revealed: the emotion of “fear” was rated as higher by observers on the right hemiface than on the left hemiface (right chimeras > left chimeras) while an opposite bias was reported for “sadness”, which registered significantly higher scores in the dogs’ left hemiface (left chimeras > right chimeras). In addition, emotion category × experimental conditions × chimeric faces interaction (F(4, 4644) = 5.703, *P* < 0.001; GLMM analysis) was revealed: pairwise comparisons showed higher scores for “happiness” at the baseline condition than in response to the approach of the unfamiliar human for both the right and left-hemiface chimeras (*P* < 0.001); a reversed result was observed for “fear” emotion, which had higher scores during the presence of the arousing stimulus than at the baseline for both chimeras (*P* = 0.001). As for “anger”, higher scores were observed during the presence of the arousing stimulus than at the baseline but only in the left-hemiface chimeras (*P* < 0.001). No other significant comparisons were identified (*P* > 0.005).

**Figure 8:**
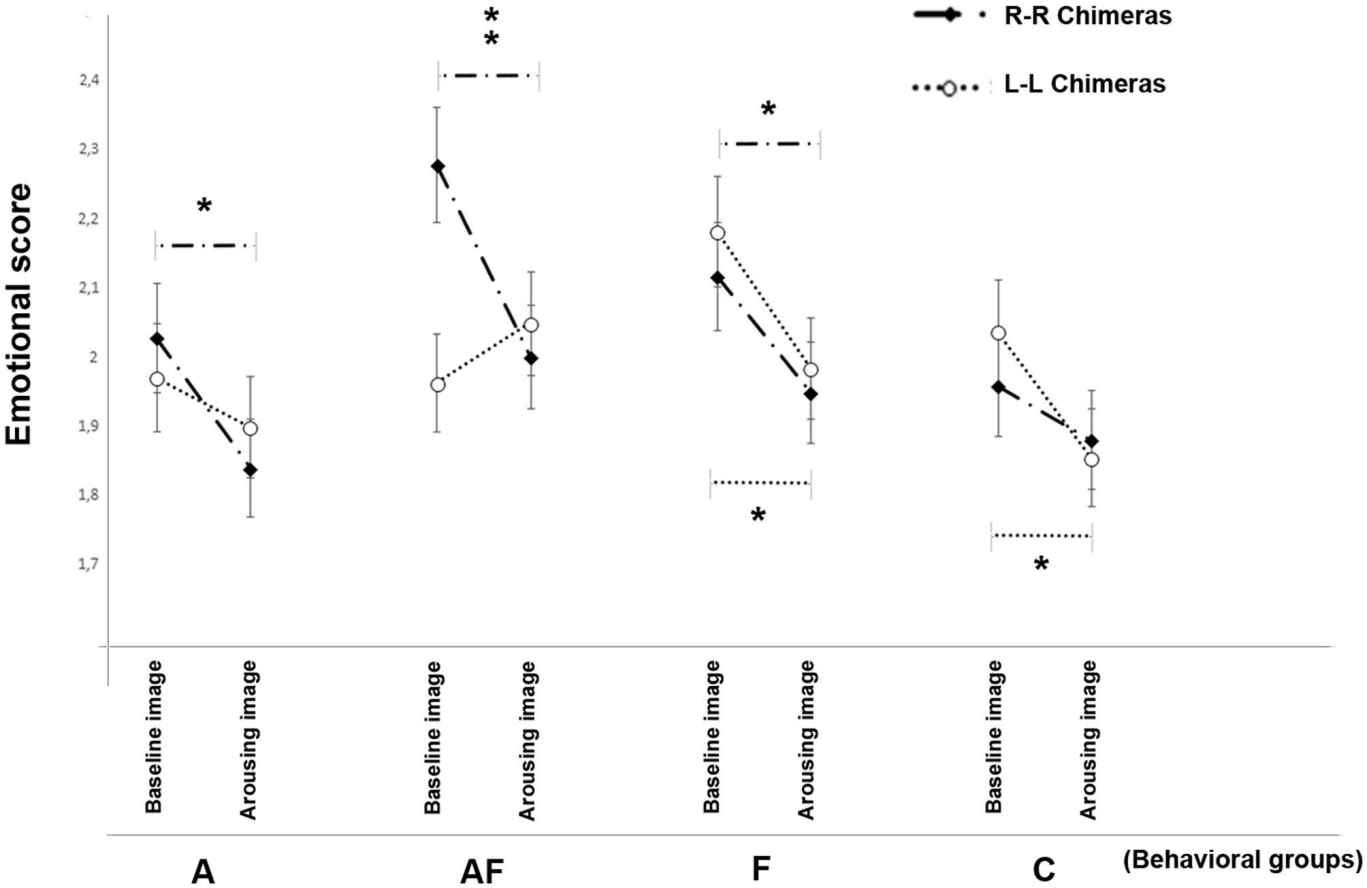
Emotional score (3). Cumulative dog facial expressions rated by humans of LL and RR chimeric faces of each behavioral group (“A”: dogs displaying high scores in stranger-directed aggression; “A–F”: dogs displaying high scores in both stranger-directed aggression and stranger-directed fear; “F”: dogs displaying high scores in stranger-directed fear; “C”: control dogs displaying low scores in both stranger-directed aggression and stranger-directed fear) during the baseline and arousing experimental conditions (group means with SEM are shown; Asterisks indicate significant biases. *P < 0.05; **P < 0.01; Fisher’s LSD test).

A statistically significant interaction between experimental conditions, chimeric faces and behavioral groups was identified (F(3, 4644) = 3.782, *P* = 0.010; Fig. 8): pairwise comparisons revealed higher scores at the baseline condition with respect to the presence of the unfamiliar human in the right-hemiface chimeras in all behavioral groups (“A” (*P* = 0.011); “F” (*P* = 0.027); “A–F” (*P* = 0.001)), with the exception of the control group (*P* = 0.260). However, left-hemiface chimeras had higher scores overall with respect to right-hemiface chimeras during the resting condition compared with the presence of the arousing stimulus in the “F” and “C” behavioral groups (*P* = 0.011).

A statistically significant interaction between the emotion category, chimeric faces and behavioral groups was revealed (F(12, 4644) = 2.376, *P* = 0.005) indicating that the emotion of “fear” was rated higher by observers on the right hemiface with respect to the left for the “A–F” group (*P* < 0.001) while an opposite bias was reported for “sadness” (“A–F”: left vs. right hemiface chimeras (*P* = 0.025)).

**Figure 9:**
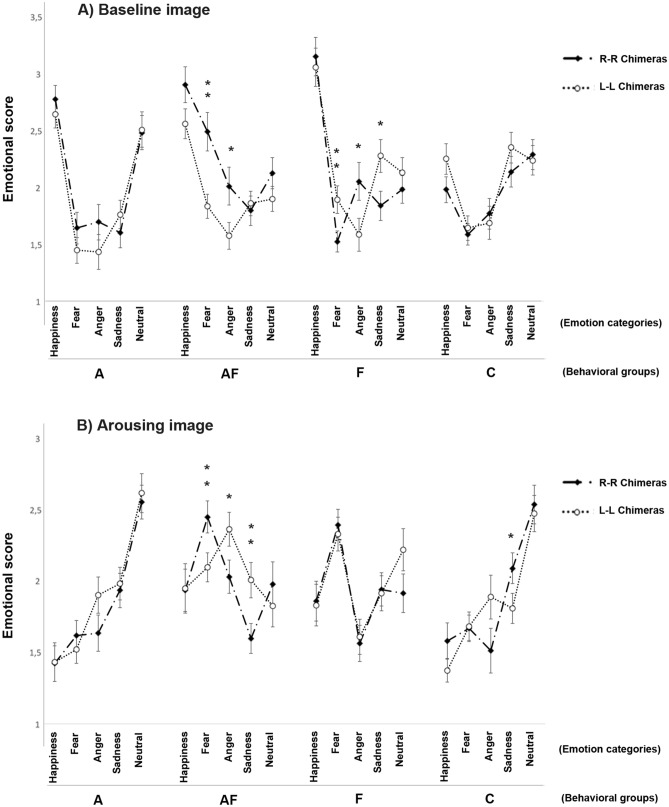
Emotional score (4). Dog facial expressions rated by humans of LL and RR chimeric faces for each emotional category (i.e. happiness, fear, anger, sadness, and neutral) of each behavioral group (“A”: dogs displaying high scores in stranger-directed aggression; “A–F”: dogs displaying high scores in both stranger-directed aggression and stranger-directed fear; “F”: dogs displaying high scores in stranger-directed fear; “C”: control dogs displaying low scores in both stranger-directed aggression and stranger-directed fear) during the baseline and arousing experimental conditions. Images taken during the baseline (**A**) and arousing (**B**) experimental conditions (group means with SEM are shown; Asterisks indicate significant biases. *P < 0.05; **P < 0.01; Fisher’s LSD test).

Finally, the emotion category × chimeric faces × behavior groups interactions at baseline ((F(12, 4644) = 19.580, *P* < 0.001) and in the presence of the arousing stimulus (F(46, 4644) = 3.706, *P* < 0.001) are shown in Fig. 9: pairwise comparisons revealed that, when dogs were alone in the presence of the owner (baseline condition; Fig. 9A), the right-hemiface obtained higher scores both for “fear” (*P* = 0.001) and “anger” (*P* = 0.022) with respect to left-hemiface chimeras in the “A–F” group; on the other hand, the “F” group registered higher scores for “fear” (*P* = 0.006) and “sadness” (*P* = 0.015) in the left-hemiface chimeras compared to the right, while an opposite bias was observed for “anger” (right-hemiface > left-hemiface chimeras, *P* = 0.025). When approach by the unfamiliar human (Fig. 9B), the facial expression of the “A–F” group obtained higher scores for “fear” (*P* = 0.006) in their right-hemiface chimeras with respect to the left, while an opposite bias was observed for the scores of both “anger” (*P* = 0.024) and “sadness” (*P* = 0.004) (left > right hemifaces chimeras). Finally, the “C” group registered higher scores for “sadness” for the right-hemiface chimeras than the left (*P* = 0.027). No other statistically significant effects were observed in score values: experimental conditions × behavioral groups (F(3, 4644) = 0.443, *P* = 0.722); chimeric faces (F(1, 4644) = 0.287, v0.592); chimeric faces × behavioral groups (F(3, 4644) = 2.321, *P* = 0.073); experimental conditions × chimeric faces (F(1, 4644) = 2.688, *P* = 0.101).

## Discussion

Our results demonstrate that dogs displaying fear and aggressive behaviors toward unfamiliar humans have higher facial asymmetries compared to the control group. The degree of such asymmetries differed between the two emotional conditions; in particular, it was higher when subjects were presented with an unfamiliar human (arousing condition) than when they were alone with their owner (baseline condition), suggesting that these asymmetries are not merely an extension of morphological asymmetries in the dogs’ faces. This result confirms previous findings regarding the presence of a significant lateralization of facial muscle movements in response to emotional stimuli in dogs (avoidance-eliciting stimuli, e.g. non-social objects)^15^. Facial asymmetries in the expression of emotions have been also reported in other mammals, including monkeys and great apes^3,25,26^. Interestingly, we report for the first time a striking effect of antisocial behaviors on dog facial asymmetries. Specifically, dogs displaying both aggressive and fearful behaviors towards unfamiliar humans (namely the “A–F” group) showed the highest level of asymmetry, followed by subjects that displayed only fear (“F” group) and aggressive (“A” group) behaviors towards unfamiliar humans respectively. Broadly speaking, facial asymmetry has been reported as an indicator of psychological, emotional, and physiological distress^17,18^ in humans. It has also been detected in those suffering from mental disorders (e.g. Autism spectrum disorder (ASD)^19^ and schizophrenia^18^), which cause impairments in the way in which they perceive and socialize with others. Similarly, dogs belonging to the “A–F” group showed disruptive social behaviors that significantly affected social interactions and their communication with humans. Indeed, the presence of a social and arousing stimulus such as the unfamiliar human significantly amplifies the asymmetry in facial expressions in the “A–F” dogs, confirming the hypothesis that the alteration in the symmetry of the face is a phenomenon related to the emotional functioning of the brain and not merely morphological or structural features. Human emotional ratings revealed significant differences in terms of emotional expressiveness in the two hemifaces of the dogs belonging to the “A–F” group. In particular, in the presence of the unfamiliar human, the right hemiface expressed the emotion of fear more than the left hemiface while, at the same time, the latter expressed more anger and sadness than the right hemiface. The simultaneous presence of these emotions would confirm the increased asymmetry detected through the calculation of the measured ratio and could also justify the disruptive behaviors of these subjects that were observed when approached by an arousing social stimulus (i.e. the unfamiliar human). The high scores registered by the questionnaire for both aggression and fear would further confirm the presence of the disruptive/antisocial behavior of these dogs toward humans. An interesting hypothesis would be that the simultaneous presence of highly arousing emotional states in the dogs’ brain would create a sort of emotional “short-circuit” which would subsequently lead to the potential expression of antisocial aggressive behavior^27,28^. It would be of interest within future studies to investigate through the use of neuro-imaging techniques whether the corpus callosum in the brain of these dogs is smaller than normal, a phenomenon which would justify the lower inter-hemispheric communication that underlies other mental disorders reported in humans^29^. It seems possible that scarce lateralization of brain functions may be associated with intense emotional responses to a broad spectrum of stimuli. One possible way of inhibiting an intense emotional response to stressful stimulus (i.e. the presence of the unknown human being in our study) would be that of shifting attention to another, less disturbing stimulus, and, from research on vision in chicks^30^ and motor lateralization in dogs^31^, it seems that lateralized neural pathways are able to do this more successfully than non-lateralized ones. The antisocial-disruptive behavior of this group could also be partially explained in the light of both “valence” and “approach-withdrawal” models. In this group, in both baseline and arousing conditions, there is a greater facial expression score for the emotion of fear in the composite photographs obtained from the right half of the dogs’ faces (RR chimera → left hemisphere). This factor would indicate that in these subjects there may also be a different functioning of the brain areas responsible for processing highly arousing emotional states as, in the animal kingdom, the emotion of fear is mainly processed by the right brain hemisphere (and not by the left), which processes negative emotions (valence model) and elicits withdrawal behavioral responses (approach-withdrawal model)^1^. In “A–F” subjects, our data suggests a prevalence for processing the emotion of fear in the left hemisphere which is, however, specialized in the analysis of positive emotions (emotional valence model) and in eliciting approach-behavioral responses (approach-withdrawal model). The misalignment of “A–F” dogs from these aforementioned models could contribute to explaining aggression (reaction of approach → left hemisphere) guided by an emotional state of fear (which should instead have elicited a withdrawal behavioral response driven by the right hemisphere). This hypothesis could confirm the concept that aggression in the dog is not a unitary concept^32^, and that the expression of aggressive behavior is closely related to a motivational balance between anger (offensive aggression) and fear (defensive aggression) emotions as has been previously shown in different breeds^33^. Furthermore, it is of interest to note that in “A–F” subjects there is a reversal in the higher intensity of the expression of anger from the RR chimera (left hemisphere) during basic conditions to the LL chimera (right hemisphere) during the arousing condition. Very recent fMRI studies mapping the spatiotemporal architecture of the whole human brain revealed that dynamic changes in brain lateralization correlate with cognitive performance^34^. Specifically, this work demonstrates that although moderate fluctuations in brain laterality over time correlated with better cognitive performance, extreme changes in laterality (i.e. “Laterality Reversal” indicating that functional laterality switches from being more ipsilateral to highly contralateral) correlated negatively with language function and cognitive flexibility, hampering cognition. The reversal laterality observed by our data in “A–F” dogs could, in the light of these new data, help explain the antisocial behavior of these subjects accompanied by very scarce cognitive flexibility and represents an element that should be taken into consideration in the future for a more complete approach to the study of laterality applied to behavioral alterations.

High level of asymmetries in dog facial expressions was also found in the fear “F” group. One of the most relevant studies supporting our results showed that asymmetry and laterality of specific facial landmarks in the resting state were associated with anxiety in a young human population^35^. Similarly, the high level of facial asymmetry registered in the dogs belonging to the “F” group during baseline conditions could be explained by the high arousal baseline level associated with anxiety in fearful dogs^36^ suggesting that even in the absence of social arousing stimuli, this emotion can cause a state of readiness that may guide dog behavioral responses to external stimuli. Furthermore, although dogs were released and let free to move and explore the testing area in order to become familiar with their environment, the possibility that fearful subjects may have been experiencing fear throughout the test due to their new surroundings cannot be ruled out. Indeed, from a visual inspection of Fig. 5B, it appears that the facial asymmetry of fearful dogs during the baseline condition was higher than in the other behavioral groups. The human judgment of the emotions expressed by the right and left chimeras further supports the results of the measured asymmetries of facial expressions in the “F” group: in particular, during baseline conditions, facial expressions were reported as expressing fear more intensely in the left-hemiface (right-hemisphere) than the right-hemiface (left hemisphere), which instead expressed anger more intensely that the left, indicating a simultaneous possible activation of different emotional areas in the dog brain. These results are in line with both the “emotional valence” and “approach-withdrawal” models. Indeed, in human studies a greater expression of negative emotions such as fear and sadness (emotions evoked from memories of past experiences) in the chimeras derived from the left hemifaces (right hemisphere) has been reported. Furthermore, the prevalent role of the right hemisphere in processing negative emotions and eliciting withdrawal behaviors has long been identified in several animal models^1^. It is of interest to note that even in human studies, the only exception to the hypothesis of the “emotional valence” of the so-called negative emotions concern the emotion of anger, which was expressed more by chimeric faces obtained from the right side of the face (left hemisphere) when humans were instructed to recall an actual experience that has elicited anger emotion and re-experience it^37^. This apparent contradiction could be explained by the approach-withdrawal model: despite being a negative emotion, anger would typically elicit aggressive behaviors that involve approach and not withdrawal reactions. On the other hand, when dogs were approached by an unfamiliar human, no significant differences in their emotional expression between the two hemifaces were detected by human subjects. This decrease in the level of facial asymmetry as reported by humans could be explained by the fact that in the presence of a social arousing stimulus, both cerebral hemispheres would prevail in the fearful stimulus analysis (facial expressiveness would follow in the same direction, attenuating the facial asymmetry) and dogs would thus employ an escape coping strategy.

Finally, although the aggressive dogs showed higher facial asymmetry than the control group, it was less pronounced than the “A–F” and “F” groups. Similar results have been reported in human studies highlighting a negative association between facial asymmetries and anger in male adolescents^17^. The recalibrational theory of anger^38^ proposes that males with a higher fighting ability, such as male adolescents in the study of Muñoz-Reyes et al.^17^, will have more power in negotiatiating for better treatment due to their greater capacity to inflict costs, deploying anger more readily^39^ as an alternative strategy (compared to physical fighting) in competing with other members of their social group. In a very similar way, the dogs in our study belonging to the aggressive group might use aggression as a coping strategy in order to ward off social stimuli. However, we cannot exclude that aggression in this group may be related more to frustration (motivated to approach) than to fear (motivation to avoid).

Overall, our results showed higher facial symmetries in dogs displaying fear and aggressive behaviors towards humans compared to the control group. Furthermore, facial asymmetries are increased in the presence of a social arousing stimulus (i.e. an unfamiliar human). This study provides further evidence of the association between brain lateralization (and lateralized behaviors) and emotional reactivity in dogs. Indeed, in previous studies, although the distribution, direction or strength of the lateralized motor behavior (in the form of paw preference) was not related to the occurrence of behavioral disorders, a significant relationship between the severity of canine behavioral problems and motor bias has been reported. In particular, increasing right-pawedness has been associated with higher scores on the C-BARQ subscale of stranger-directed aggression and stranger-directed fear in dogs showing behavioral issues^40^. Furthermore, the use of paw preference as a marker for emotionality in dogs has been recently challenged by Simon and colleagues, who found no correlations between paw preference tests and emotional reactivity scales (measured by Positive and Negative Activation Scale, PANAS)^41^. These findings rise methodological questions about the assessment of paw preference and emotionality in dogs, indicating that motor asymmetries might be task-specific and have variable task-consistency.

In this light, future studies could investigate the potential relationship between task-specific asymmetries of motor behaviors and emotional facial expressions in dogs. Moreover, measuring facial asymmetries in dogs could prove to be a useful non-intrusive investigative tool in evaluating possible physiology-based behavioral disorders suggesting the use of facial expression analysis in clinical and behavioral veterinary science.

## Supplementary Information

Supplementary Information.

## Data Availability

The datasets generated and/or analyzed during the current study are available from the corresponding author on reasonable request.
