# Supplementary material for: Facial asymmetry in dogs with fear and aggressive behaviors towards humans
Source: Sci Rep. 2022 Nov 15;12:19620. doi: 10.1038/s41598-022-24136-2 (PMC9666627; doi:10.1038/s41598-022-24136-2)
Supplement: Supplementary file 1 — Supplementary Information. [file 41598_2022_24136_MOESM1_ESM.docx]

**Supplementary Information**

**Title of manuscript:**

"Facial asymmetry in dogs with fear and aggressive behaviors towards humans”.

**Authors:**

Marcello Siniscalchi*, Serenella d’Ingeo, Michele Minunno & Angelo Quaranta

Animal Physiology and Behavior Unit, Department of Veterinary Medicine, University of Bari Aldo Moro, 70121 Bari, Italy.

*Corresponding author: Marcello Siniscalchi, Animal Physiology and Behavior Unit, Department of Veterinary Medicine, University of Bari Aldo Moro, 70121 Bari, Italy; e-mail: marcello.siniscalchi@uniba.it

**Supplementary Table S1.** Questionnaire presented to owners in order to gather information about their dogs’ temperament [1].

| Item |
| --- |

*1-Stranger-directed aggression*

Dog acts aggressively

When approached directly by and unfamiliar male adult while being walked or exercised on a leash

When approached directly by and unfamiliar female adult while being walked or exercised on a leash

When approached directly by and unfamiliar child while being walked or exercised on a leash

Toward unfamiliar persons approaching the dog while it is the owner’s car

When an unfamiliar persons approaching the owner or a member of the owner’s family at home

When an unfamiliar persons approaching the owner or a member of the owner’s family away from home

When mailmen or other delivery workers approach the home

When strangers walk past the home while the dog is in the yard

When joggers, cyclists, roller skateboarders pass the home while the dog is in the yard

Toward unfamiliar persons visiting the home

*2-Owner-directed aggression and fear*

Dog acts aggressively

When verbally corrected or punished by a member of the household

When toys, bones, or other objects are taken away by a member of the household

When bathed or groomed by a member of the household

When approached directly by a member of the household while it is eating

When food is taken away by a member of the household

When stared at directly by a member of the household

When a member of the household retrieves food or objects stolen by the dog

*3-Stranger-directed fear*

Dog acts anxious or fearful

When approached directly by an unfamiliar male adult while away from the home

When approached directly by an unfamiliar female adult while away from the home

When approached directly by an unfamiliar child adult while away from the home

When unfamiliar persons visit the home

**Supplementary Table S2.** Summary of the significant effects of different factors on the "Facial asymmetry index” and the “Emotional score” variables.

|  | **Facial asymmetry index** | | | **Emotional score** | | |
| --- | --- | --- | --- | --- | --- | --- |
|  | F | (df1, df2) | P | F | (df1, df2) | P |
| **Experimental conditions** (presence of the owner alone/ approach of an unfamiliar human being) | 9.919 | (1,24) | 0.004 | 25.878 | (1,4644) | 0.000 |
| **Behavioral groups**  (“A”, “F”, “A-F” and “C”) | 53.359 | (1,24) | 0.000 | 8.333 | (3,4644) | 0.000 |
| **Emotion category**  (happiness, fear, anger, sadness, and neutral) | - | - | - | 44.866 | (4,4644) | 0.000 |
| **Chimeric faces**  (R-R chimera and L-L chimera) | - | - | - | - | - | - |
| **Experimental conditions x Behavioral groups** | 13.978 | (3,24) | 0.000 | - | - | - |
| **Emotion category x Experimental conditions** | - | - | - | 65.683 | (4,4644) | 0.000 |
| **Emotion category x Behavioral groups** | - | - | - | 19.580 | (12,4644) | 0.000 |
| **Emotion category x Experimental conditions x Behavioral groups** | - | - | - | 6.983 | (12,4656) | 0.000 |
| **Emotion category x Chimeric faces** | - | - | - | 2.865 | (4,4644) | 0.022 |
| **Emotion category x Experimental conditions x Chimeric faces** | - | - | - | 5.703 | (4,4644) | 0.000 |
| **Experimental conditions x Chimeric faces x Behavioral groups** | - | - | - | 3.782 | (3,4644) | 0.010 |
| **Emotion category x Chimeric faces x Behavioral groups** | - | - | - | 2.376 | (12,4644) | 0.005 |

**Reference**

1. Hsu, Y. & Serpell, J. A. Development and validation of a questionnaire for measuring behavior and temperament traits in pet dogs. J. Am. Vet. Med. Assoc. 223, 1293-1300 (2003).
